# Dependency on *de novo *protein synthesis and proteomic changes during metamorphosis of the marine bryozoan *Bugula neritina*

**DOI:** 10.1186/1477-5956-8-25

**Published:** 2010-05-24

**Authors:** Yue Him Wong, Shawn M Arellano, Huoming Zhang, Timothy Ravasi, Pei-Yuan Qian

**Affiliations:** 1 Department of Biology, Hong Kong University of Science and Technology, Clear Water Bay, Hong Kong SAR; 2 The King Abdullah University of Science and Technology (KAUST), The Kingdom of Saudi Arabia

## Abstract

**Background:**

Metamorphosis in the bryozoan *Bugula neritina *(Linne) includes an initial phase of rapid morphological rearrangement followed by a gradual phase of morphogenesis. We hypothesized that the first phase may be independent of *de novo *synthesis of proteins and, instead, involves post-translational modifications of existing proteins, providing a simple mechanism to quickly initiate metamorphosis. To test our hypothesis, we challenged *B. neritina *larvae with transcription and translation inhibitors. Furthermore, we employed 2D gel electrophoresis to characterize changes in the phosphoproteome and proteome during early metamorphosis. Differentially expressed proteins were identified by liquid chromatography tandem mass spectrometry and their gene expression patterns were profiled using semi-quantitative real time PCR.

**Results:**

When larvae were incubated with transcription and translation inhibitors, metamorphosis initiated through the first phase but did not complete. We found a significant down-regulation of 60 protein spots and the percentage of phosphoprotein spots decreased from 15% in the larval stage to12% during early metamorphosis. Two proteins--the mitochondrial processing peptidase beta subunit and severin--were abundantly expressed and phosphorylated in the larval stage, but down-regulated during metamorphosis. MPPbeta and severin were also down-regulated on the gene expression level.

**Conclusions:**

The initial morphogenetic changes that led to attachment of *B. neritina *did not depend on *de novo *protein synthesis, but the subsequent gradual morphogenesis did. This is the first time that the mitochondrial processing peptidase beta subunit or severin have been shown to be down-regulated on both gene and protein expression levels during the metamorphosis of *B. neritina*. Future studies employing immunohistochemistry to reveal the expression locality of these two proteins during metamorphosis should provide further evidence of the involvement of these two proteins in the morphogenetic rearrangement of *B. neritina*.

## Background

To explain the rapid metamorphosis of many marine invertebrates, a 'need for speed' hypothesis has been suggested due to strong predation pressures and the relative defenselessness of transitioning larvae [1]. To cope, the larvae of many marine invertebrates enable rapid metamorphosis by the pre-formation of some juvenile structures [1,2]. Metamorphosis of the cosmopolitan marine bryozoan *Bugula neritina*--a species that causes biofouling problems worldwide [3-5] and also yields the antitumor compound bryostatin [6]--completes very rapidly and the morphological changes associated with metamorphosis have been described well [7,8]. However, the presumptive juvenile tissues in the larvae of *B. neritina *are largely undifferentiated when they commit to metamorphosis [8]. In this case, we suggest the rapidity of metamorphosis in *B. neritina *may be facilitated by a simple and quick mechanism that is independent of *de novo *protein synthesis and instead involves post- translational modification of existing proteins.

In *B. neritina*, metamorphosis can be divided into two phases. The first phase of metamorphosis is dramatic and quick; metamorphosis initiates when the swimming larva attaches to the substratum, with concomitant morphogenetic movements to internalize the larval ciliated epithelium and form the precursor to the cystid (juvenile body wall) and polypide (the lophophore and digestive tract). The first phase completes within minutes and transforms the swimming larva into a sessile, transitory metamorphic stage termed the "preancestrula." The second phase of metamorphosis is more gradual, ranging in duration from 36 to 48 h and includes complete degradation of larval tissues and substantial morphogenesis such as the elongation of the tubular preancestrula, differentiation of the polypide, and complete development of the cystid, resulting in a juvenile or "ancestrula" [5,6]. Whether the two phases of metamorphosis in *B. neritina *correlate with a shift from independence to dependence on *de novo *protein synthesis is unknown.

A recent study that compared the phosphoproteomic and proteomic changes associated with metamorphosis of *B. neritina *suggested that metamorphosis may rely on phosphorylation of existing proteins rather than *de novo *synthesis of proteins [9]. It was then proposed that several phosphoproteins were de-phosphorylated during metamorphosis of *B. neritina *[9]. However, the lack of a comprehensive protein database for *B. neritina *hindered their identification of the de-phosphorylated proteins. Moreover, phosphoproteomic and proteomic changes during early metamorphosis were not closely monitored. Since the morphological changes and the rapidity of metamorphosis vary drastically as metamorphosis of *B. neritina *proceeds, the *B. neritina *proteome may also change between its two phases of metamorphosis. We hypothesized that the first phase of metamorphosis in *B. neritina *involves post-translational modifications of existing proteins and is independent of *de novo *protein synthesis, while the second phase of metamorphosis depends on *de novo *protein synthesis. Thus, we expected different phosphoproteome patterns between the swimming larvae and the newly attached preancestrulae (2 h) of *B. neritina*, and different total proteome patterns between the newly attached preancestrulae (2 h) and the further developed preancestrulae (4 h).

To test our hypotheses, we examined metamorphosis of *B. nertina *in the presence of transcription and translation inhibitors. We found that swimming larvae initiated but could not complete metamorphosis in the presence of either inhibitor. We then employed 2DE- based proteomics to characterize changes in the phosphoproteome and the total proteome in the swimming larvae and two stages (2 h preancestrulae and 4 h preancestrulae) during the second phase of metamorphosis of *B. neritina*. Proteins that were phosphorylated, de- phosphorylated, or differentially expressed were identified using LC-MS/MS. Finally, we performed qRT-PCR to analyze the gene expression pattern of the identified proteins.

## Methods

### Collection of *Bugula neritina *adults and larvae

**Figure 1:**
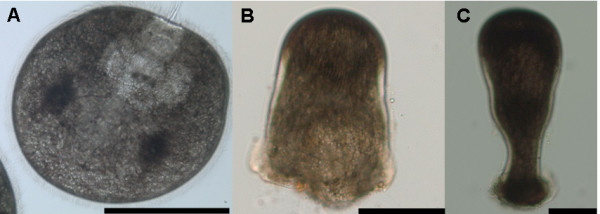
**Developmental stages of *Bugula neritina *selected for proteome/phosphoproteome mapping**. (A) Swimming larvae immediately after release from adult, (B) 2 h preancestrulae, sampled 1 h after larval attachment, and (C) 4 h preancestrulae, sampled 3 h after larval attachment. Scale bars = 100 μm.

Adult *B. neritina *were collected from the floating rafts of a fish farm in San Shing Wan, Hong Kong (22°21'19 N, 114°16'15E) between February and April, 2009. Adult colonies were maintained in a flow-through seawater system in the Coastal Marine Laboratory at the Hong Kong University of Science and Technology for no more than 7 d before use. Sexually mature *B. neritina *colonies were placed in a 10 L glass tank filled with 0.45 μm filtered seawater under bright artificial light to induce release of larvae from the brood chambers of adult colonies. Swimming larvae were carefully selected under a dissecting microscope and were immediately used for experiments, sampled for proteomic analysis, or allowed to metamorphose and further develop to the given stages for proteomic analysis (Fig. 1). Newly released *B. neritina *larvae reduce their light-positive swimming behavior and initiate attachment and metamorphosis in high synchrony without the addition of a chemical inducer.

### Transcription and translation inhibitor experiments

To determine whether the initiation of metamorphosis in *B. neritina *is transcription/translation independent, we incubated newly released larvae with either the transcription inhibitor DRB or the translation inhibitor emetine (Sigma Alrich, St. Louis, MO, USA) and observed metamorphosis in comparison with controls. DRB and emetine are stable in seawater and have been shown to block up to 90% of *de novo *protein synthesis in the larvae of the red abalone *Haliotis rufescens *and the polycheate *Hydroides elegans *[10,11]. Between 40 and 70 larvae were added to the wells of 24-well plates (Becton Dickinson, Franklin Lakes, NJ, USA) containing 2 ml of AFSW plus one of the inhibitors (pH 8.2). Treatments consisted of six concentrations of each inhibitor (0.01, 0.1, 1, 10, 20, and 40 μM dissolved in DMSO), plus two controls--one containing 1% DMSO in AFSW and one containing AFSW only. There were 4 replicates for each treatment. The experimental plates were kept in the dark at room temperature (around 24°C). After 1 h, the number of larvae that had initiated metamorphosis was counted under a dissecting microscope. A larva was considered to have initiated metamorphosis when it was attached to the well surface or to the water surface and its ciliated epithelium was internalized (i.e., the surface of the attached larva appeared smooth). After counting, the experimental plates were returned to the dark for an additional 40 h. At the termination of the experiments, the animals that had completed metamorphosis into ancestrulae were counted. Percent data were arcsine transformed and analyzed with a repeated measures ANOVA, with time as the repeated measure and concentration as a fixed factor, followed by a Dunnett's test using AFSW as the control treatment.

To check if the two compounds were toxic to *B. neritina *larvae, after 40 h of inhibitor treatment, the inhibitor solutions were replaced with AFSW and the larvae were returned to the dark for an additional 40 h. Then, the larvae that had resumed metamorphosis were counted. The resumption of metamorphosis was signified by the elongation of the preancestrulae or development of the polypide. The percentage of *B. neritina *that resumed metamorphosis was calculated and arcsine transformed prior to a one-way ANOVA analysis. Finally, we assessed the effectiveness of the inhibitors by measuring the length of the metamorphosing individuals in the presence of each inhibitor over time. Swimming larvae were treated with the minimum effective concentration of either DRB or emetine (0.1 μM and 10 μM, respectively). Mean lengths of 5 metamorphosing individuals were measured at 1, 4, 8, 20, 28 and 40 h.

### 2DE sample preparation

Three stages were sampled for proteomic analysis (Fig. 1). 'Swimming larvae' were newly released and processed immediately. In general, newly released *B. neritina *larvae become competent to metamorphose after a brief swimming period, ranging from a few minutes to several hours [12]. Two stages ('2 h preancestrulae' and '4 h preancestrulae') were sampled during the second phase of metamorphosis. The 2 h preancestrulae had internalized the ciliated epithelium (i.e., the surfaces of the attached larvae appeared smooth; Fig 1B) and the 4 h preancestrulae had also substantially elongated their tubular bodies and had degraded most larval tissues (Fig. 1C). Comparisons between the swimming larvae and 2 h preancestrulae should reveal proteomic changes necessary to initiate and complete the first phase of metamorphosis (i.e., attachment) and initiate morphogenesis. Comparisons between the 2 h preancestrulae and 4 h preancestrulae should reveal proteomic changes associated with morphogenesis (i.e., during the second phase of metamorphosis).

Swimming larvae were concentrated on a mesh sieve, washed with AFSW followed by PBS, and then immediately fixed in lysis buffer consisting of 7 M urea, 2 M thiourea, 4% CHAPS, 1% DTT, 1× phosphatase inhibitor (PhosSTOP; Roche, Basel, Switzerland), and 1× protease inhibitor (cOmplete; Roche, Switzerland). To obtain metamorphosing *B. neritina *samples, we added newly released larvae to a petri-dish containing 10-ml AFSW and allowed them to initiate metamorphosis in the dark for 1 h. After 1 h of incubation, unattached larvae were discarded and metamorphosing individuals were allowed to develop for an additional 1 or 3 h (2 h preancestrulae and 4 h preancestrulae, respectively). The 2 h preancestrulae and 4 h preancestrulae were collected by scraping off individuals using an autoclaved glass cover slip. The samples were then washed with PBS twice and transferred to lysis buffer for 2DE. Three batches of samples from each stage were used.

Extraction procedures followed those in Thiyagarajan et al. [9] except that sonicated samples were first incubated in 30°C water bath for 10 min and then centrifuged at 15,000 g for 10 min before the supernatant, containing the total soluble protein fraction, was transferred to a new 2-ml tube. The 2DE cleanup kit (BioRad, Hercules, CA, USA) was used to purify our protein samples. The purified protein pellets were rehydrated in rehydration buffer consisting of 7 M urea, 2 M thiourea, 4% CHAPS, 40 mM DTT, 0.5% 4-7 ampholyte (Biolyte; BioRad, USA), and 1% bromophenol blue, and incubated for 15 min in a 30°C heat block with orbital shaking to enhance protein solubilization. Protein concentration was determined using an RC/DC protein assay kit (BioRad, USA).

### 2D Gel Electrophoresis

Since most of the phosphoproteins in *B. neritina *larvae and juveniles have an acidic p*I *value [9], we performed IEF using pH 4-7 linear 17 cm IPG strips (BioRad, USA) in an attempt to increase the resolution. The IPG strips were loaded with 300 μg of proteins and then actively rehydrated at 50 V for 14 h. IEF was performed under the following condition: 100 V for 30 min, 500 V for 1 h, 1000 V for 2 h, followed by a gradient to 8000 V until it reached a total of 60000 Vh. The electric current during IEF did not exceed 50 μA per strip. Before the second dimensional SDS-PAGE, the strips were equilibrated for 10 min in reducing equilibration buffer I containing 6 M Urea, 0.05 M Tis-HCl (pH 8.8), 2% SDS, 50% glycerol, 2% (w/v) DTT, and then 10 min in alkylating equilibration buffer II containing 2.5% (w/v) iodoacetamide instead of DTT. Next, the reduced and alkylated strips were used for 12% SDS-PAGE (2,000 mm × 2,000 mm × 1 mm). The running buffer system was the standard Laemmli buffer for SDS-PAGE. Protein standard was the broad range protein marker (Takara, Dalian, China). The gels were run at 20°C at a maximum of 24 mA per gel until the phenol blue dye reached the bottom of the gel. To ensure repeatability between gel runs, 2DE was repeated twice with the same amount of protein and under the same running conditions for each replicate of each stage.

The 2DE gels were fluorescently stained with Pro-Q Diamond phosphoprotein gel stain (Invitrogen, Carlsbad, CA, USA) by fixing the gels in a solution of 40% methanol/10% acetic acid overnight, washing three times with deionized water for 20 min each time, incubating in Pro-Q Diamond stain for 150 min, and then destaining with successive washes in 20% ACN in 50 mM sodium acetate (pH 4.0). After 3 h, the gels were imaged using a TYPHOON scanner (GE Heathcare Life Science, Piscataway, NJ, USA) at 532 nm excitation and with a 610 BP 30 emission filter. Following image acquisition, the gels were stained overnight for total proteins with Sypro Ruby protein gel stain (Invitrogen, USA) and then scanned using 582 nm excitation and 610 BP 30 emission filters.

### Gel analysis

The gel images from both the phosphoprotein fluorescent stain and total protein stain were analyzed using PDQuest software (version 8.0; BioRad, USA) according to the protocol provided by the software developer. The PDQuest software filtered and smoothed each gel by determining the maximum absorption after correction of the raw image and background subtraction creating filtered images. The software then fit the protein spots in the filtered image to a three-dimensional Gaussian distribution model, creating the Gaussian image. Spot detection was undertaken using the spot detection wizard on the Gaussian image with the largest spot and faintest spot defined based on visual inspection. Only the spots that were consistently detected in all three replicates and two repeats were counted as positive detections. The images were aligned with land marking to ensure that corresponding protein spots were matched. We overlaid the gel images representing the phosphoproteome and total proteome to ensure correct matching of phosphoprotein spots to the corresponding spots in the total proteome.

To analyze the total proteome and the phosphoproteome, a Pro-Q diamond stained gel match set and a Sypro Ruby-stained gel match set were created; match sets allowed the comparison between replicate gels and gels from different stages. Spot intensities were normalized to make the total density in each gel image equal. The analysis was performed in quantitative and qualitative modes. The quantitative detection of changes in protein abundance was set at a threshold of more than 2 fold. Spots that were significantly different (Student-t test, *p *< 0.01) between successive stages were also considered. Protein spots were defined as up- or down-regulated only when they showed more than 2-fold difference in intensity and were significantly different between successive stages.

### Protein identification by LC-MS/MS Mass Spectrometry

To facilitate protein spot excision, 2D gels were stained with Coomassie blue G250. Protein spots of interest from the preparative gels were excised and destained with 100 mM NH_4_-HCO_3 _and 50% methanol. After the destaining buffer was removed, the gel pieces were dried in a vacuum centrifuge and rehydrated in 10 μL of trypsin digestion reaction mix containing 10 mM NH_4_HCO_3_, 10% ACN, and 50 ng of trypsin (sequencing grade; Promega, Fitchburg, WI, USA). After digestion overnight at 37°C, the peptides were extracted twice with 5% formic acid in 50% ACN. The extracts were pooled together and dried in a vacuum centrifuge. The dried digest was reconstituted in 10 μL of 0.1% formic acid in water. The sample was concentrated in a peptide trap (Waters, Milford, MA, USA) and then analyzed using a nanoflow UPLC (nanoAcquity™, Waters, USA) coupled with an ESI Q-TOF tandem mass spectrometer (Premier™, Waters, USA). The peptide separation was performed in a BEH130 C18 analytical column (75 μm ID × 25 cm, 1.7 μm particles; Waters, USA). Mobile phase A (0.1% formic acid in water) and mobile phase B (0.1% formic acid in ACN) were used to establish a 90 min gradient comprised of 5% mobile phase B for 3 min, 5-30% mobile phase B for 47 min, followed by 30-80% mobile phase B for 15 min and 80% mobile phase B for 10 min, and finally re-equilibrated at 5% mobile phase B for 15 min. The UPLC was operated at a constant flow rate of 200 nL min^-1^. The ion source was set at capillary voltage 2.4 kV, source temperature 80°C, sample cone voltage 35 V, and collision cell gas flow rate was 0.50 mL min^-1^. The lock mass was sampled every 30 s. The Q-TOF was set to perform data-dependent acquisition in the positive ion mode with a selected MS survey mass range of 300-1600 *m/z*. The abundant peptides with +2 to +4 charge states above a 40 count threshold were selected for MS/MS. The time of summation of MS/MS events was set to 3 s and dynamically excluded for 10 s with ± 30 mmu mass tolerance.

The raw MS/MS data was converted into .pkl file using ProteinLynx (v2.2.5, Waters, USA) (smooth 3/2 Savitzky Golay and center 4 channels/80% centroid) and merged using Mascot daemon (v 2.2) prior to Mascot search against our customized *Bugula neritina *database (submitted to NCBI, accession number SRA010777.2) [13]. The database contains 13,863 protein sequences in total. Enzyme limits were set at full tryptic cleavage at both ends; a maximum of two missed cleavages was allowed. Mass tolerances were set at 20 ppm for peptide precursors and 0.5 Da for fragment ions. Carboxamidomethylation (+57.02) at cysteine residues was set as fixed modification; oxidation (+15.99) at methionine, phosphorylation at serine, threonine or tyrosine (+79.96) were set as variable modifications. Proteins with at least two significant peptide hits (*p *< 0.05) were reported as positive identification.

### cDNA synthesis and semi-quantitative real time PCR

Total RNAs from the swimming larvae, 2 h preancestrulae, and 4 h preancestrulae were isolated using the TRIzol reagent (Invitrogen, USA), according to the supplier's instructions. The extracted total RNAs were digested with DNase (Turbo DNA-free™ Kit, Applied Biosystems, Foster City, CA, USA) to remove trace DNA contaminants. The cDNA was synthesized from 2 μg of total RNA from each stage using M-MLV reverse transcriptase (USB, Cleveland, OH, USA) with random hexamer primer. Gene specific primers were designed based on nucleotide sequence of the target protein in the *B. neritina *database. The *B. neritina *18S RNA gene [14] was chosen as the reference gene for normalizing the expression levels of target genes. The details of the primers are in Table S1 of additional file 1. qRT-PCR assays for each target gene were performed in triplicate and repeated twice. All qRT-PCR assays were carried out using iTaq SYBR Green Supermix with ROX (BioRad, USA) and were run on the Stratagene mx3000p PCR machine (Agilent Technologies, Santa Clara, CA, USA). The qRT-PCR data were analyzed by the 2^-ΔΔCT ^Method according to Livak and Schmittgen [15].

## Results

### Transcription and translation inhibitor experiments

**Figure 2:**
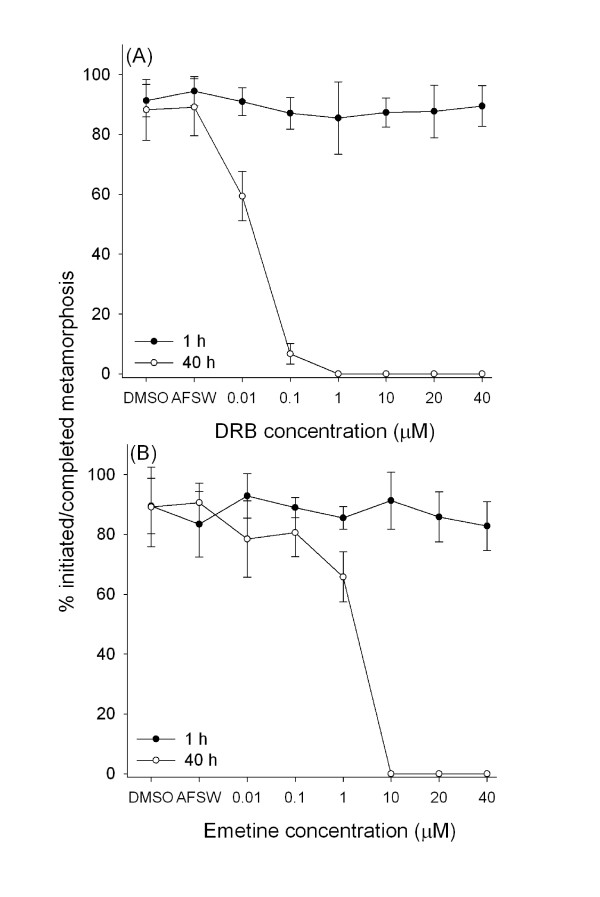
**Percent of *Bugula neritina *initiated or completed metamorphosis in the presence of various concentrations of (A) the transcription inhibitor DRB and (B) translation inhibitor emetine after 1 h (solid circles) and 40 h (open circles)**. At 1 h, individuals that had initiated metamorphosis were counted. At 40 h, only those individuals that had completed metamorphosis (i.e., developed to ancestrulae) were counted. AFSW and DMSO served as controls. Error bars are + 1 S.D., n = 4.

**Figure 3:**
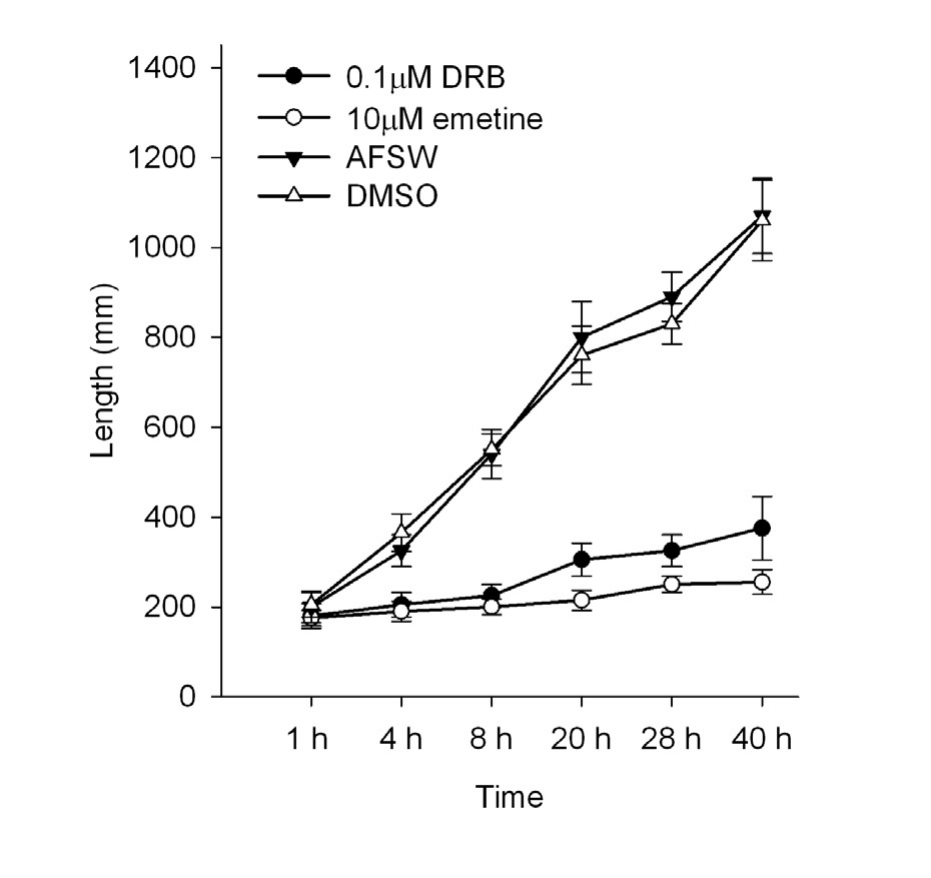
**Elongation of metamorphosing *Bugula neritina *(preancestrulae) in the presence of DRB (0.1 μM) or emetine (10 μM) and in control (AFSW and DMSO)**. Error bars are + 1 S.D, n = 5.

In the presence of the transcription inhibitor DRB and the translation inhibitor emetine, the swimming larvae of *B. neritina *attached and initiated metamorphosis up to the preancestrulae stage, but the subsequent phase of metamorphosis was inhibited at most concentrations. After 1 h in either inhibitor at any concentration, the percentages of individuals that had initiated metamorphosis in the treatment and the controls were not significantly different (Dunnett's test: *p *> 0.05 for all contrasts with AFSW) (Fig. 2). For both inhibitors, concentration significantly affected the percentage of individuals completing metamorphosis at 40 h (F = 47.61, *p *< 0.001) (Fig. 2). For DRB, all tested concentrations significantly inhibited completion of metamorphosis when compared to AFSW (Dunnett's test: *p *= 0.012) (Fig. 2A). For emetine, concentrations ≥ 1 μM significantly inhibited completion of metamorphosis in comparison to the individuals in AFSW (Dunnett's test: *p *= 0.01) (Fig. 2B). Although the two inhibitors blocked the completion of metamorphosis within 40 h, over this time period, we did observe slower metamorphosis, as indicated by the elongation of the preancestrulae, of individuals placed in either 0.1 μM DRB or 10 μM emetine than individuals in the controls (AFSW or DMSO) (Fig. 3). There was no mortality observed for either inhibitor in any of the tested concentrations within the experimental duration. Metamorphosis blocked by all the concentrations of either DRB or emetine was resumed and completed within another 40 h with 100% recovery in all tested concentrations.

### Dynamic changes in phosphoproteome and total proteome

**Figure 4:**
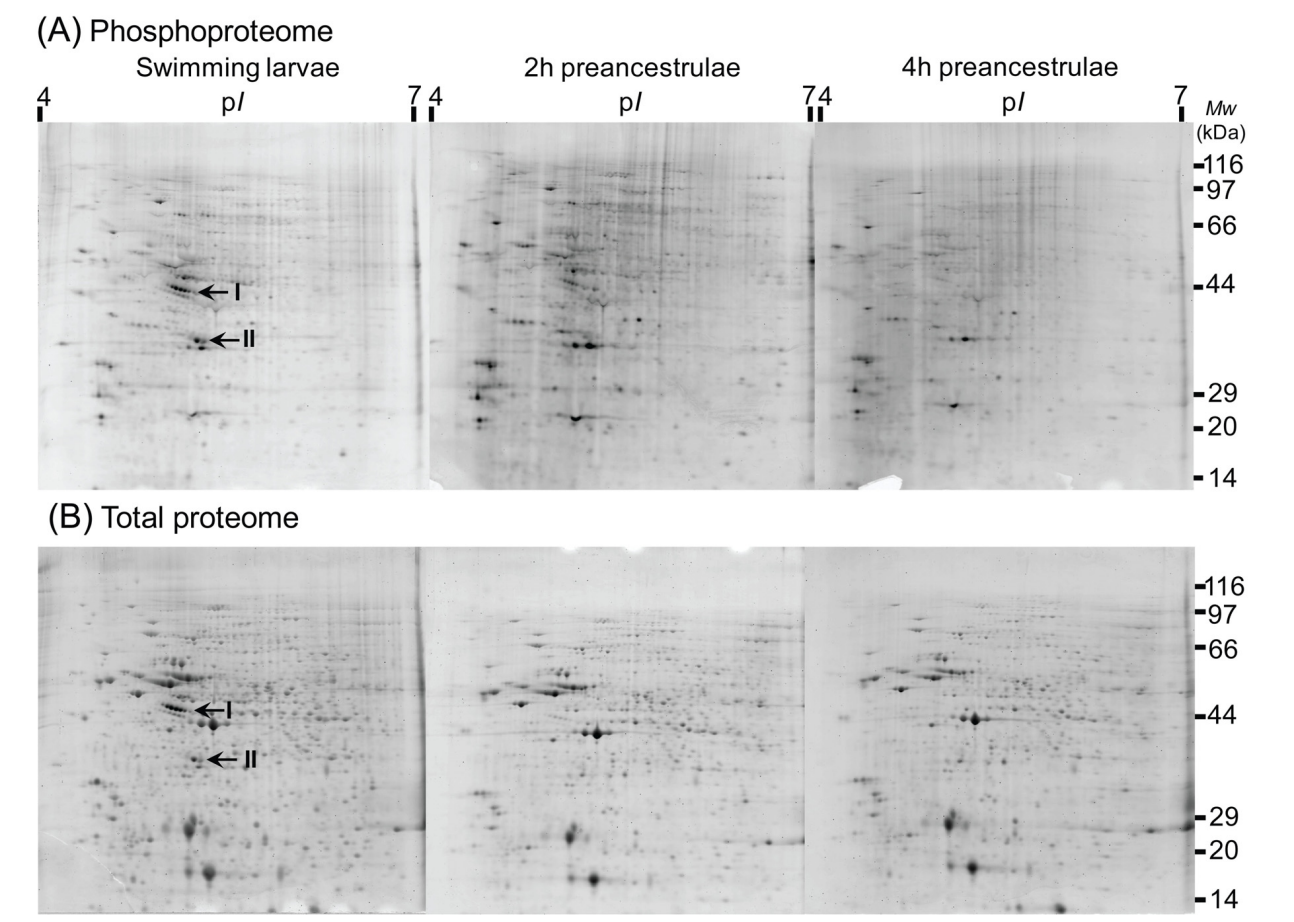
**Representative 2DE gels stained with (A) phosphoprotein-specific dye, Pro-Q Diamond, and (B) total protein dye, Sypro-Ruby, in swimming larvae, 2 h preancestrulae, and 4 h preancestrulae**. Total protein samples (300 μg) were separated on linear IPG strips (p*I *4-7) followed by 12% SDS-PAGE. The results presented here are representative of 2 repeats of 3 replicates for each sample. Arrows indicate areas I and II magnified in Figure 6.

**Figure 5:**
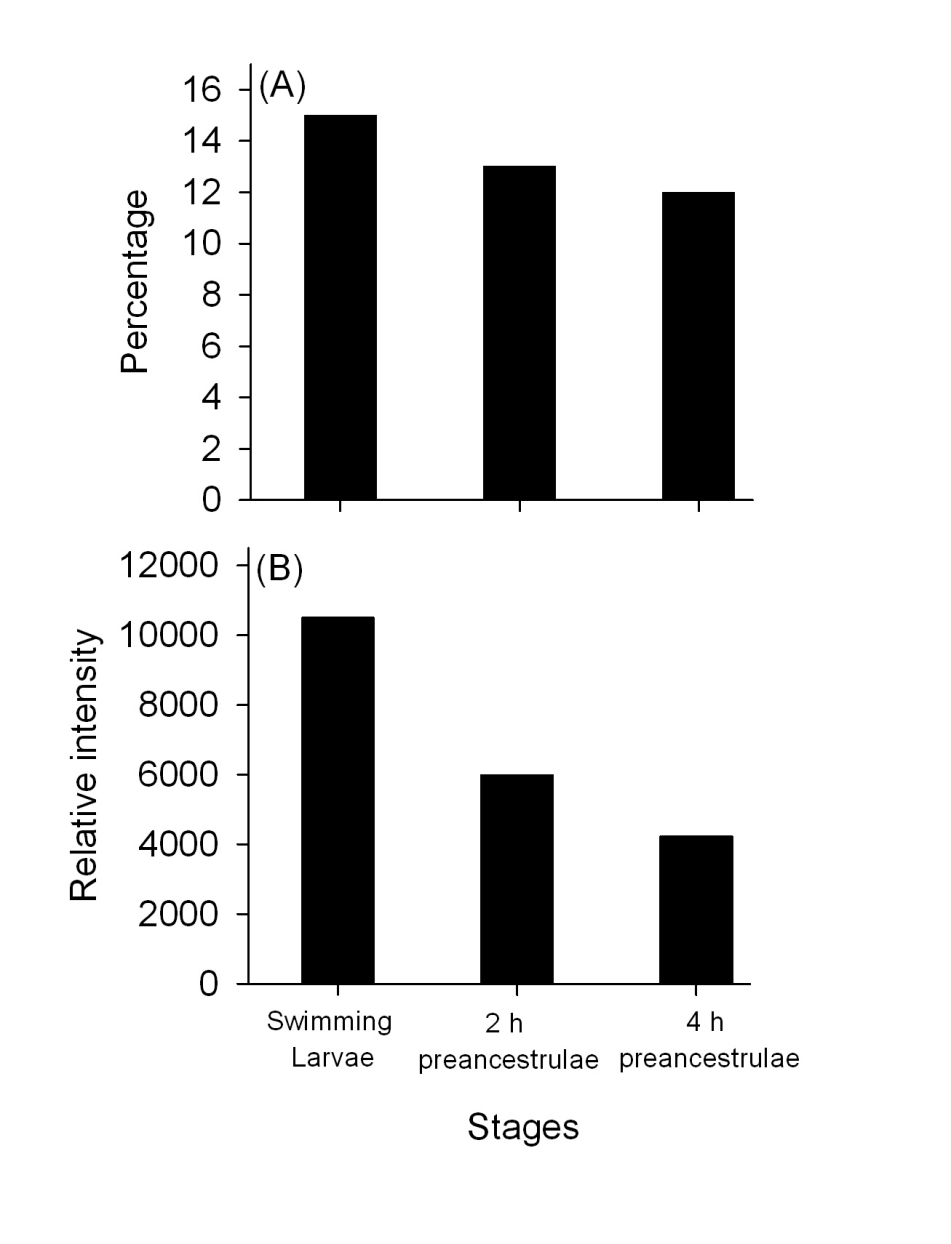
**Detailed analysis of the phosphoproteome of successive stages**. (A) Percentage of protein spots that were phosphorylated and (B) the overall intensity of the phosphoproteome, which is the summation of the relative intensity of all the phosphoprotein spots at one stage.

Representative 2D gels stained with either phosphoprotein specific stain or total protein stain are shown in Figure 4. We reproducibly detected 81, 69, and 64 phosphoprotein spots and 526, 520, and 519 protein spots in all replicate gel images of the swimming larvae, 2 h preancestrulae, and 4 h preancestrulae, respectively. All the phosphoprotein spots from the different stages were mapped to the corresponding protein spots from the corresponding stage. The percentage of detected phosphorylated protein spots decreased from about 15% in the swimming larvae to 13% in 2 h preancestrulae and 12% in 4 h preancestrulae (Fig. 5A). The overall intensities of the phosphoproteomes of 2 h preancestrulae and 4 h preancestrulae were 2-fold and 2.5-fold less than in the swimming larvae, respectively (Fig. 5B).

Forty protein spots were down-regulated between the swimming larvae and the 2 h preancestrulae. Twenty proteins spots were down-regulated between the 2 h preancestrulae and 4 h preancestrulae. Neither up-regulation of protein spots nor any stage-specific protein spots between the swimming larvae and the 2 h preancestrulae were detected; the same was true between the 2 h preancestrulae and 4 h preancestrulae.

**Figure 6:**
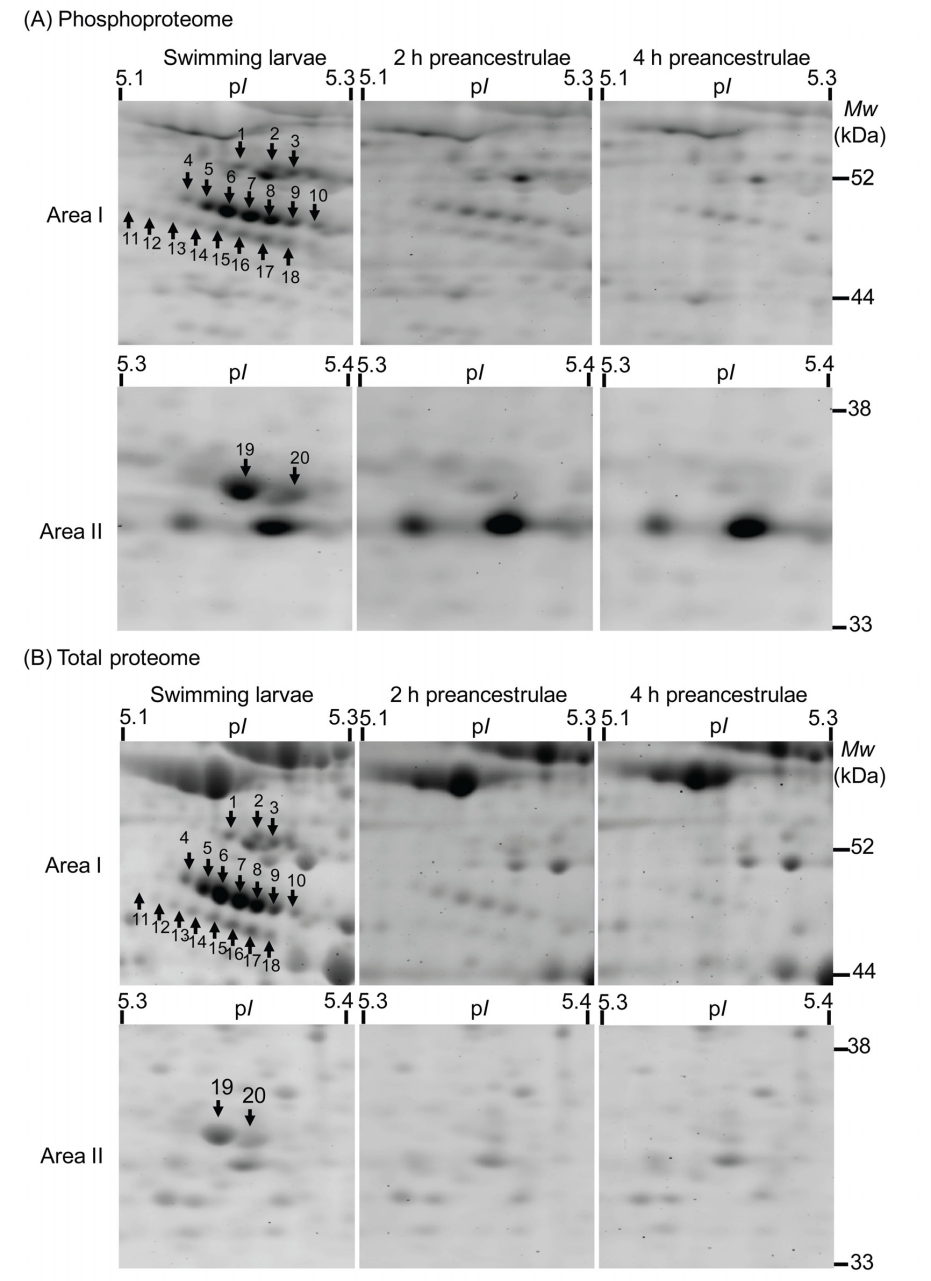
**A close view of selected phosphoproteins (indicated by arrows) altered during early metamorphosis**. (A) Two different areas (I & II) of the phosphoproteome were selected for each developmental stage. (B) The corresponding total protein 2DE gel for Area I and Area II. The 2DE gels presented here are representatives of 2 repeats of 3 replicates for each metamorphic stage.

**Figure 7:**
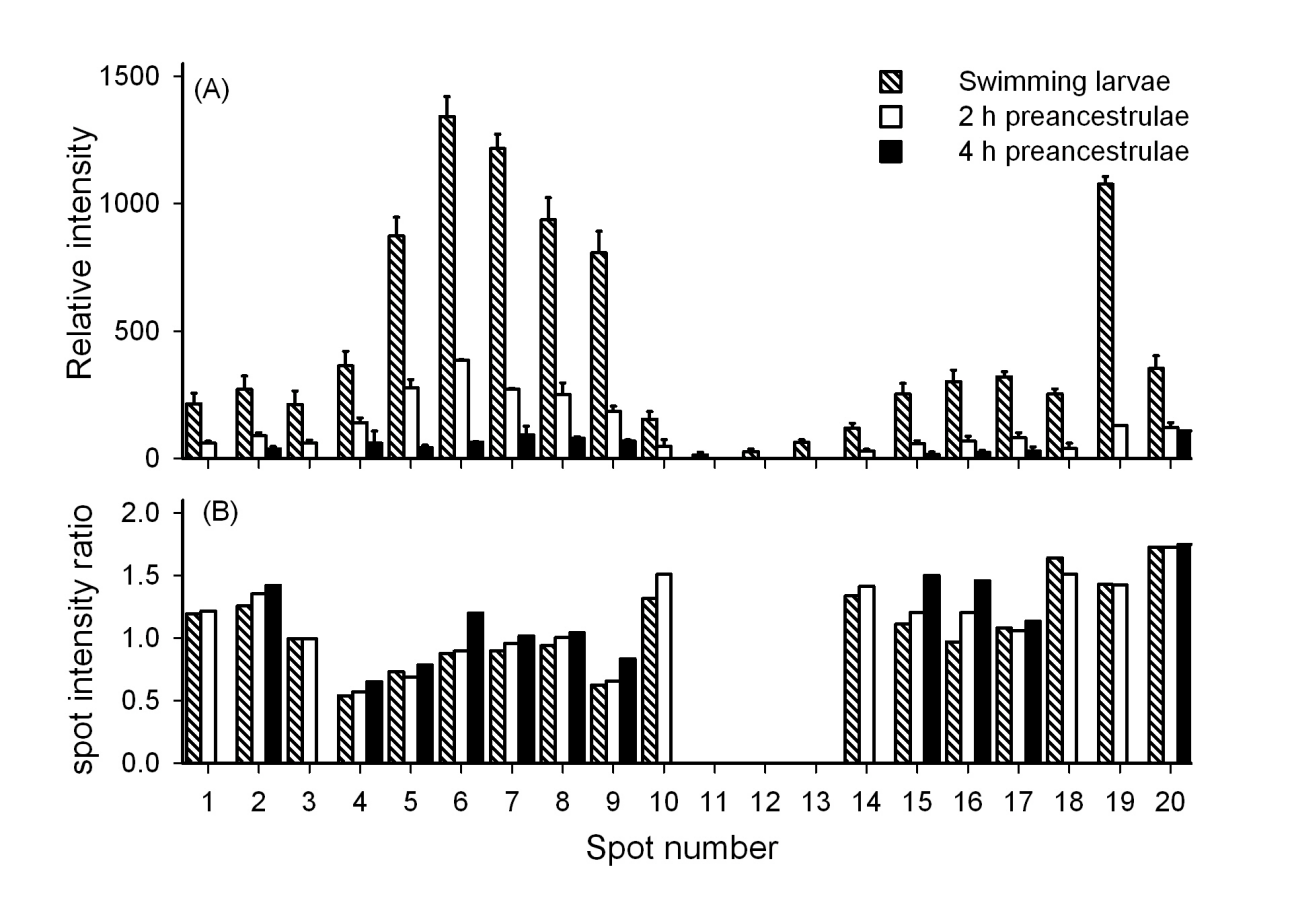
**(A) Intensities of selected protein spots and (B) their phosphoprotein:protein spot intensity ratios**. Analyzed spots were significantly different (*p *< 0.01) and showed more than 2 fold variation between the three developmental stages. Spots are shown in Figure 6.

Among those differentially expressed proteins spots, 20 protein spots that were phosphorylated and abundantly expressed in the swimming larvae, but drastically down- regulated in the 2 h preancestrulae and 4 h preancestrulae, were analyzed further. These 20 protein spots are indicated in Figure 6, which shows magnified images of two specific areas that are indicated by arrows in Figure 4. All 20 spots were down-regulated by at least 3 fold; spots 11, 12, and 13 were not detectable in the 2 h preancestrulae. All detectable spots, except spot 20, were further down-regulated by >2-fold in the 4 h preancestrulae; spots 1,3,14,18, and 19 were not detectable in the 4 h preancestrulae (Fig. 7A). We suspected that spot 1-10 and 14-20 may be de-phosphorylated in the preancestrulae, in addition to down-regulated. To determine whether they were de-phosphorylated, the phosphoprotein:protein relative spot intensity ratios of these 17 spots were compared in all the three stages. For any protein de- phosphorylation, the phosphoprotein relative spot intensity should decrease more drastically than the protein relative spot intensity, resulting in decreasing phosphoprotein:protein relative spot intensity ratios between successive stages. However, the phosphoprotein:protein relative spot intensity ratios increased or stayed constant between successive metamorphic stages (Fig. 7B), suggesting that these 20 phosphoprotein spots were down-regulated rather than de- phosphorylated.

### Identification of differentially expressed proteins by mass spectrometry

Among the 20 selected spots, spot 1, 2, and 3 were too weak to be detected by LC- MS/MS and the mass spectra of spots 19 and 20 did not match to any entry in the *B. neritina *protein database. The remaining 15 spots were confidently identified (p < 0.01) (Table 1; Figure S1 of additional file 2). Spots no. 4-10 (p*I *5.15-5.28; MW 47-51 kDa) matched well to the same gene, the putative mitochondrial processing peptidase beta subunit (MPPbeta). Spots no. 11-18 (p*I *5.1-5.26; MW 45-49 kDa) also matched to a single gene, severin, a Ca^2+^- dependent F-actin fragmentation protein.

**Table 1:** Protein identification by LC-MS/MS of spot no. 4-18 indicated in Figure 6.

Spot no.	Protein identified	Accession. No.	Mascot score (no. of unique peptide)	MW (kDa)/p*I*	Unique peptide	Miss cleavage	Score	Expect
4	the putative mitochondrial processing peptidase, beta subunit	SRA010777.2, Contig1413_30	237(3)	50.7/5.15	RIPLAELDAR	1	56	2.7e-006
					AMGGGANLPSILASK	0	101	1.6e-010
					LCNNVTNFEVQR	0	86	4.7e-009
5	the putative mitochondrial processing peptidase, beta subunit	SRA010777.2, Contig1413_30	311(5)	49.8/5.17	IPLAELDAR	0	52	0.0005
					RIPLAELDAR	1	62	3.4e-005
					AMGGGANLPSILASK	0	68	3.1e-005
					LCNNVTNFEVQR	0	64	0.0001
					AVGPVEQLDDYGILR	0	61	0.00016
6	the putative mitochondrial processing peptidase, beta subunit	SRA010777.2, Contig1413_30	349(3)	49.1/5.19	RIPLAELDAR	1	60	9.8e-007
					AMGGGANLPSILASK	0	78	3.3e-008
					LCNNVTNFEVQR	0	86	4e-009
7	the putative mitochondrial processing peptidase, beta subunit	SRA010777.2, Contig1413_30	546(4)	48.4/5.21	IPLAELDAR	0	68	2.1e-007
					RIPLAELDAR	1	56	2.8e-006
					AMGGGANLPSILASK	0	106	5e-011
					LCNNVTNFEVQR	0	80	1.3e-008
8	the putative mitochondrial processing peptidase, beta subunit	SRA010777.2, Contig1413_30	976(5)	47.7/5.23	IPLAELDAR	0	61	1.1e-006
					RIPLAELDAR	1	64	3.9e-007
					AMGGGANLPSILASK	0	93	8e-010
					LCNNVTNFEVQR	0	82	9.2e-009
					IESVDAQAVMNVCTK	0	77	2.8e-008
9	the putative mitochondrial processing peptidase, beta subunit	SRA010777.2, Contig1413_30	1154(5)	47.1/5.25	IPLAELDAR	0	59	1.5e-006
					RIPLAELDAR	1	58	1.6e-006
					AMGGGANLPSILASK	0	90	1.5e-009
					LCNNVTNFEVQR	0	100	1.5e-010
					IESVDAQAVMNVCTK	0	84	6.3e-009
10	the putative mitochondrial processing peptidase, beta subunit	SRA010777.2, Contig1413_30	525(4)	47/5.28	IPLAELDAR	0	59	1.2e-006
					RIPLAELDAR	1	62	5.7e-007
					AMGGGANLPSILASK	0	114	7.4e-012
					LCNNVTNFEVQR	0	94	5.5e-010
11	Severin	SRA010777.2, Contig4181_4	262(4)	49.1/5.1	LVYMDGGVASGFR	0	65	3.7e-007
					YNAAAYCQQLESER	0	58	1.4e-006
					TDVLEEDSTPETHEFYEK	0	88	1.6e-009
					TVELDTYHNDGPVQHR	0	52	1.1e-005
12	Severin	SRA010777.2, Contig4181_4	389(4)	48.4/5.12	LVYMDGGVASGFR	0	61	1e-006
					YNAAAYCQQLESER	0	78	1.7e-008
					TVELDTYHNDGPVQHR	0	52	1.1e-005
					TDVLEEDSTPETHEFYEK	0	105	3.1e-011
13	Severin	SRA010777.2, Contig4181_4	445(4)	47.7/5.14	LVYMDGGVASGFR	0	61	1e-006
					YNAAAYCQQLESER	0	78	1.7e-008
					TVELDTYHNDGPVQHR	0	52	1.1e-005
					TDVLEEDSTPETHEFYEK	0	105	3.1e-011
14	Severin	SRA010777.2, Contig4181_4	404(4)	47.1/5.16	LVYMDGGVASGFR	0	54	6.3e-006
					YNAAAYCQQLESER	0	85	3e-009
					TVELDTYHNDGPVQHR	0	121	1.3e-012
					TDVLEEDSTPETHEFYEK	0	127	2e-013
15	Severin	SRA010777.2, Contig4181_4	340(3)	46.5/5.19	LVYMDGGVASGFR	0	68	2.1e-007
					YNAAAYCQQLESER	0	87	2e-009
					TDVLEEDSTPETHEFYEK	0	96	2.8e-010
16	Severin	SRA010777.2, Contig4181_4	1013(7)	45.9/5.21	EVQAHESSLFK	0	78	2e-008
					IYQFNGANCSK	0	57	2.3e-006
					LVYMDGGVASGFR	0	77	2.4e-008
					MDFELVAEGTFSK	0	77	2e-008
					YNAAAYCQQLESER	0	99	1.4e-010
					TVELDTYHNDGPVQHR	0	122	9.6e-013
					TDVLEEDSTPETHEFYEK	0	124	3.7e-013
17	Severin	SRA010777.2, Contig4181_4	611(5)	45.5/5.24	EVQAHESSLFK	0	52	9.8e-006
					LVYMDGGVASGFR	0	60	1.3e-006
					YNAAAYCQQLESER	0	81	7.3e-009
					TVELDTYHNDGPVQHR	0	113	8.3e-012
					TDVLEEDSTPETHEFYEK	0	122	6.8e-013
18	Severin	SRA010777.2, Contig4181_4	472(5)	45.3/5.26	EVQAHESSLFK	0	57	0.0006
					HSTQDEYGTAAYK	0	94	1.3e-007
					YNAAAYCQQLESER	0	79	5e-006
					TDVLEEDSTPETHEFYEK	0	112	3.1e-009
					AKTDVLEEDSTPETHEFYEK	1	154	1.6e-013

### Gene expression profiling of differentially expressed proteins

**Figure 8:**
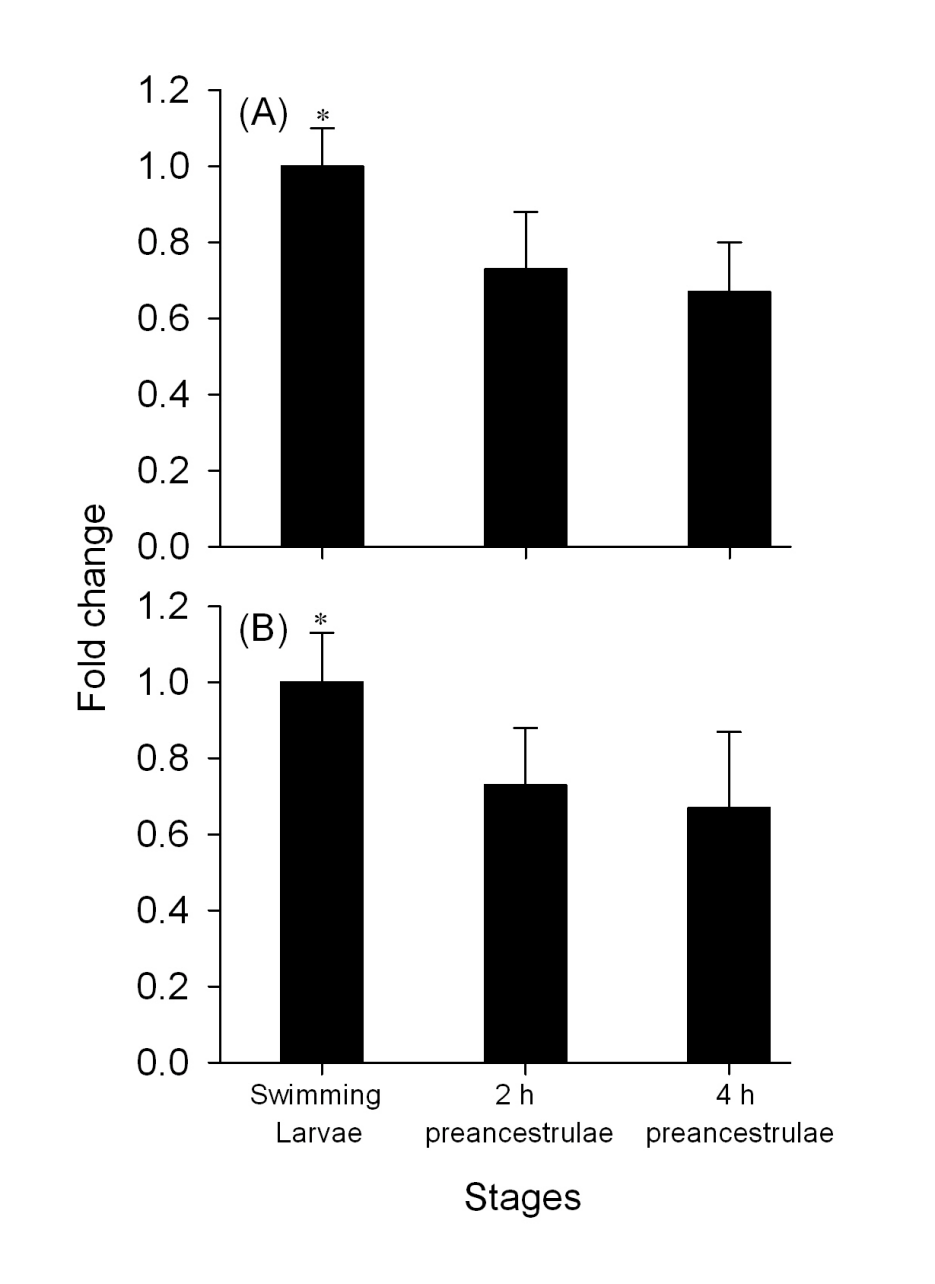
**The gene expression profile of (A) Mitochondrial processing peptidase beta subunit and (B) Severin as determined by qRT-PCR**. Significant differences (p < 0.05) as indicated by Student's t-test are indicated by *.

Compared to the level of gene expression in the swimming larvae, the expression of MPPbeta decreased by 1.5 fold in 2 h preancestrulae and by 1.7 fold in 4 h preancestrulae (p < 0.05) (Fig. 8A). The gene expression level of severin decreased by 1.9 fold in 2 h preancestrulae and 4 h preancestrulae (p < 0.05) when compared to that of the swimming larvae (Fig. 8B). Hence, both MPPbeta and severin were down-regulated on both gene and protein expression levels during early metamorphosis.

## Discussion

We hypothesized that the first phase of metamorphosis of *B. neritina*, the dramatic and rapid transformation of swimming larvae into preancestrulae, is independent of *de novo *protein synthesis, but instead involves post-translational modifications of existing proteins, while the second phase of metamorphosis, consisting of the development of preancestrula into the ancestrula, requires transcription and translation. Our results from the transcription and translation inhibitor treatments support our hypothesis. Metamorphosis of *B. neritina *was arrested after the initial morphological rearrangement when either transcriptional or translational inhibitor was present, suggesting the first phase of metamorphosis is independent of *de novo *protein synthesis. Although emetine and DRB have been shown to induce apoptosis and disrupt cell-cycle regulation leading to abnormal development in some species [16,17], we did not observe any abnormal metamorphosis in the inhibitor treatments. Moreover, the results from the recovery assays showed that the effects of emetine and DRB were temporary in *B. neritina *and metamorphosis resumed upon their removal.

Woollacott et al. [7] and Reed et al. [18] pointed out that during metamorphosis of *B. neritina*, the initial morphological rearrangement during the first phase of metamorphosis is mediated by larval muscle contraction. Shimizu et al. [5] demonstrated that biogenic amines are present in several major parts of the *B. neritina *larval nervous system, including neuronal cell bodies and fibers in the apical plate, paraxial nerve cords, neural plexus, eyespots, and the equatorial nerve ring. Hence, the nervous system may be responsible for perceiving stimulants for attachment, then initiating and coordinating the larval muscle contraction; therefore, *de novo *synthesis of new proteins may not be necessary during the first phase of metamorphosis in this species.

The second phase of metamorphosis is slower and involves substantial morphogenesis. Our results suggest that this phase depends on *de novo *synthesis of proteins; in the presence of transcription/translation inhibitors, development of preancestrulae could not proceed through to elongation and the subsequent differentiation of the cystid and polypide. But, when the inhibitors were removed, metamorphosis resumed and completed within 48 hours. We hypothesized that protein phosphorylation status may be important during the first phase of metamorphosis of *B. neritina *and the initiation of the second phase because 1) we showed that their metamorphosis could initiate, but not complete, without *de novo *synthesis of proteins, 2) settlement and metamorphosis has been suggested to be necessarily speedy [1] and changes in protein phosphorylation status serve as a rapid molecular switch in many higher organisms [19,20], and 3) in insect and amphibian metamorphosis, protein phosphorylation networks are known to undergird many developmental processes such as apoptosis during tail reabsorption of amphibian tadpoles, innate immunity, and generation of nervous systems [21-23]

We expected to see a differential pattern in the phosphoproteomes between the swimming larvae and the newly transformed preancestrulae (2 h preancestrulae) of *B. neritina*, and a differential pattern in the total proteomes between the 2 h preancestrulae and 4 h preancestrulae. Indeed, we detected fewer phosphoprotein spots and a decreased overall spot intensity in the 2 h preancestrulae and 4 h preancestrulae, indicating there were fewer phosphorylated proteins during early metamorphosis. Although we did see a differential pattern in the total proteomes between the 2 h preancestrulae and 4 h preancestrulae, we were surprised to observe only down-regulation between any of the three stages. Metamorphosis of *B. neritina *requires both morphogenesis and degeneration of larval tissue and these processes should involve activation of various cellular and metabolic pathways. Thus, we expected to observe some up-regulation in addition to down-regulation in both phosphoproteome and total proteome during this period. We believe such discrepancy may have been due to the fact that we used a narrow p*I *range (4-7) to profile the proteome/phosphoproteome, which could have missed some important proteins outside this isoelectric range. In fact, Thiyagarajan et al [9] found both up-regulation and down-regulation of phosphoprotein in later metamorphic stages of *B. neritina*, possibly because they used p*I *3-10 IPG strips. In addition, 2DE is known for its poor representation of low-abundance proteins [24,25]. Therefore, we cannot rule out the possibility that there was indeed up-regulation of low-abundance proteins that were out of the detection limit of 2DE.

In this study, we focused on 20 protein spots that were phosphorylated and abundantly expressed in the swimming larvae but drastically down-regulated in the 2 h preancestrulae and 4 h preancestrulae. We confidently identified (p < 0.01) 15 out of these 20 spots using LC-MS/MS and searching against the *Bugula neritina *customized database that we recently generated [13]. Spots 4-10 and 11-18, which appeared as two chains of protein isoforms on the 2D gels, matched to the putative MPPbeta and severin, respectively. Gene expression of MPPbeta and severin, as detected by qPT-PCR, were also down-regulated during metamorphosis, which correlated to the protein expression pattern.

Severin is a Ca^2+^-dependent F-actin fragmentation protein. Together with other actin- binding proteins, severin regulates the dynamic rearrangement of actin cytoskeleton, which is of critical importance to maintenance of cell morphology and polarity, endocytosis, and intracellular trafficking [26]. Reed et al. [18] demonstrated that the rapid morphogenetic movements in *B. neritina *are an actin microfilament-mediated process. For instance, they reported that actin filament assemblages are responsible for the complete internalization of larval ciliated epithelium and the subsequent migration of the preancestrula wall epithelium. In this study, we found that severin was abundantly expressed in the swimming larvae but down-regulated as metamorphosis of *B. neritina *progressed. We suggest that severin is intensely engaged in the actin filament-mediated morphogenetic rearrangement during the first phase of metamorphosis but is not as important during the subsequent morphogenesis phase. Additionally, the severin in *B. neritina *was phosphorylated. In *Dictyostelium *amoebae, severin is phosphorylated upon extracellular stress, leading to cytoskeleton rearrangement [27]. It is still unclear, however, how phosphorylation is related to the role of severin in metamorphosis of *B. neritina*.

The MPP is a heterodimeric, zinc-dependent metalloprotesase [28], consisting of the substrate recognizing alpha subunit and the catalytic, zinc-binding beta subunit [29-32]. The alpha/beta MPP complex is essential to the maturation of mitochondrial proteins in yeast [33] and has also been shown to be important to mitochondrial proliferation and maintenance [34]. Both alpha and beta subunits of MPP are required for cleavage activity [35]; in this study, we found down-regulation of only MPPbeta, instead of both subunits. We found seven isoforms of MPPbeta in *B. neritina *that were abundantly expressed and phosphorylated in the swimming larvae, then down-regulated in the 2 h preancestrulae and 4 h preancestrulae. Czarna et al. [36] recently reported that the amoeba *Dictyostelium discoideum *has four MPPbeta isoforms that were differentially regulated during vegetative growth, but when *D. discoideum *encountered nutritional stress and went through subsequent development, all four isoforms of MPPbeta were down-regulated. However, currently there is no documentation on phosphorylation of MPPbeta, nor on how their phosphorylation status would affect the MPP complex cleavage activities. It remains unclear whether and how the down-regulation of MPPbeta is involved in triggering morphogenesis or whether it was simply the result of morphological rearrangement in *B. neritina*. Nonetheless, this is the first time that MPPbeta was found to be drastically down-regulated during the metamorphosis of *B. neritina*. Localization of MPPbeta and other MPP related proteins by immunohistochemistry may help us unravel how down-regulation of MPP affects the morphogenetic changes during the early metamorphosis of *B. neritina*.

## Conclusions

Here we demonstrated that the initial morphogenetic changes that lead to attachment of *Bugula neritina *are not dependent on *de novo *protein synthesis but the changes in the subsequent morphogenetic phase are. The phosphoproteome of *B. neritina *changed during metamorphosis, with fewer phosphoprotein spots detected in the preancestrulae than in the swimming larvae. Surprisingly, we detected only down-regulation of protein spots in the total proteome. Among 20 spots that were phosphorylated and abundantly expressed in the swimming larvae but drastically down-regulated in the early metamorphosis, 7 spots and 8 spots matched to the putative mitochondrial processing peptidase beta subunit and the calcium-dependent, actin binding protein severin, respectively. Gene expression of MPPbeta and severin, as revealed by qRT-PCR, correlated with the protein expression pattern.

## List of abbreviations

2DE: Two dimensional gel electrophoresis; LC-MS/MS: Liquid chromatography tandem mass spectrometry; qRT-PCR: Semi-quantitative real time PCR; DRB: 5,6-dichloro-1-β-D-ribofuranosylbenzimidazole; AFSW: Autoclaved, 0.45 μm filtered seawater; DMSO: Dimethyl sulfoxide; ANOVA: Analysis of variance; PBS: Phosphate buffered saline; DTT: Dithiothreitol; IEF: Isoelectric focusing; IPG: Immobilized pH gradient; SDS-PAGE: Sodium dodecyl sulfate-polyacrylamide gel electrophoresis; ACN: Acetonitrile; UPLC: Ultraperformance liquid chromatography; ESI Q-TOF: Electrospray quadrupole-time of flight; Q-TOF: Quadrupole time of flight; MS: Mass spectrometry; MS/MS: Tandem mass spectrometry; MPPbeta: Mitochondrial processing peptidase beta subunit; MPP: Mitochondrial processing peptidase.

## Competing interests

The authors declare that they have no competing interests.

## Authors' contributions

YHW designed and carried out all experiments, data analysis, and drafted the original manuscript. SMA advised on experimental design and statistical analysis, and helped draft the original manuscript. HZ carried out protein identification by mass spectrometry and provided helpful suggestions on the data analysis and manuscript. PYQ and TR conceived and designed the broad study and advised on all aspects. All authors approved the final manuscript.

## Supplementary Material

Additional file 1 **Table S1 - The nucleotide sequences of gene specific primers of target genes**. All primers have at least 50% GC content. Click here for file

Additional file 2 Figure S1 - The MS/MS spectra of unique peptides that matched to (A) MPPbeta and (B) severin. Click here for file
