# Supplementary material for: Dependency on de novo protein synthesis and proteomic changes during metamorphosis of the marine bryozoan Bugula neritina
Source: Proteome Sci. 2010 May 24;8:25. doi: 10.1186/1477-5956-8-25 (PMC2890537; doi:10.1186/1477-5956-8-25)
Supplement: Additional file 1 — Table S1 - The nucleotide sequences of gene specific primers of target genes. All primers have at least 50% GC content. [file 1477-5956-8-25-S1.doc]

| Target protein | Accession no. | Forward primer | Reverse primer | Product length (bp) |
| --- | --- | --- | --- | --- |
| the putative Mitochondrial Processing Peptidase, beta subunit | SRA010777.2, Contig1413_30 | CCCCAGCCGTCTTCACTGGC | GAGGGAAGGTTGGCACCGCC | 181 |
| Severin | SRA010777.2, Contig4181_4 | ACTGAACCGGCTTGGGAGAA | TGGGCTGTTGGGATCAGGCT | 162 |
| 18S rRNA | AF499749 | CCGGCGACGCCTTCACTGAG | CGCGCCTGCTGCAAACCTTG | 154 |
