# Supplementary figures and images for: Dependency on de novo protein synthesis and proteomic changes during metamorphosis of the marine bryozoan Bugula neritina
Source: Proteome Sci. 2010 May 24;8:25. doi: 10.1186/1477-5956-8-25 (PMC2890537; doi:10.1186/1477-5956-8-25)

**(A)**


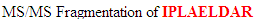


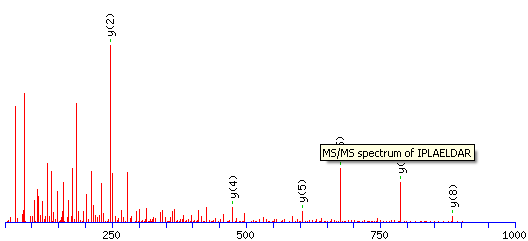


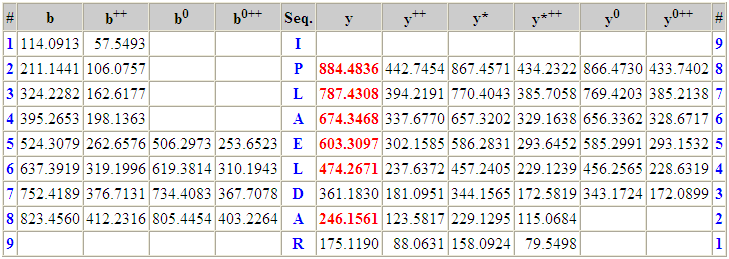


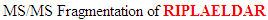


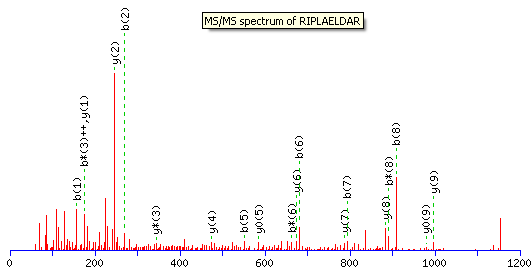


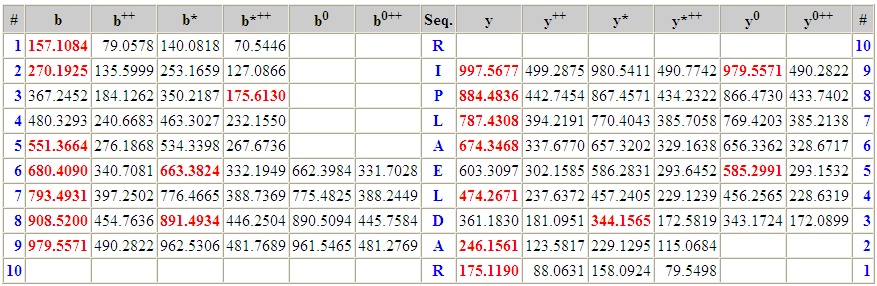


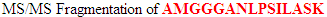

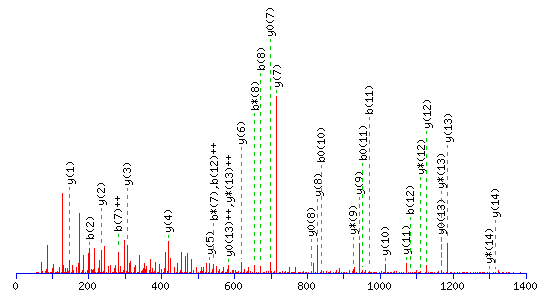


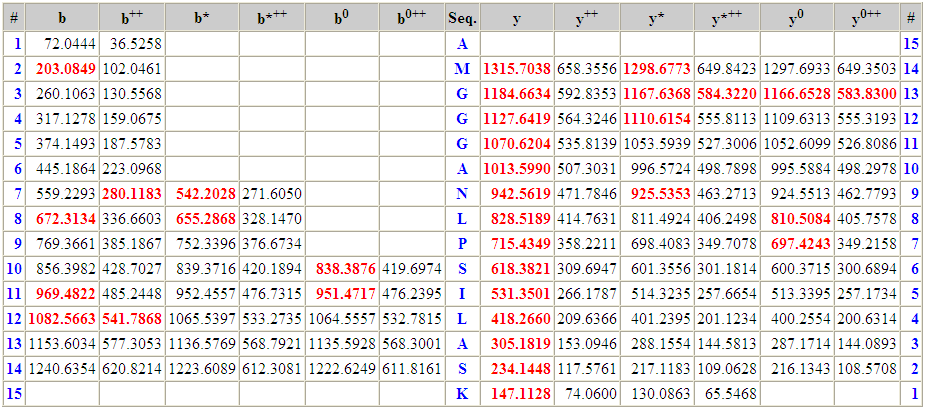


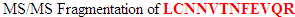


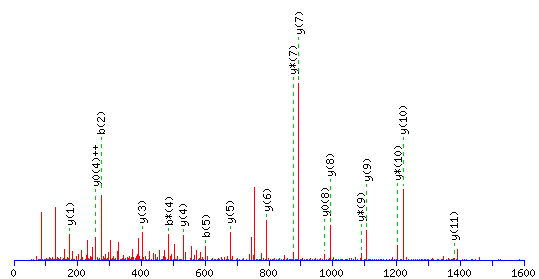


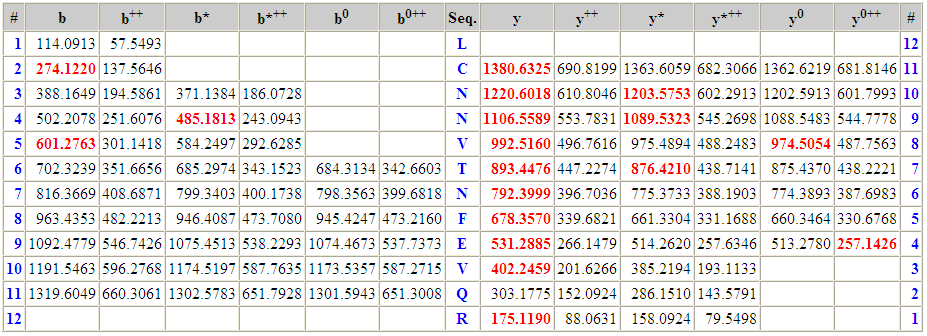


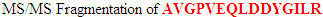


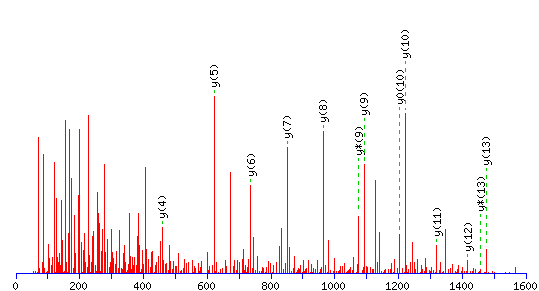


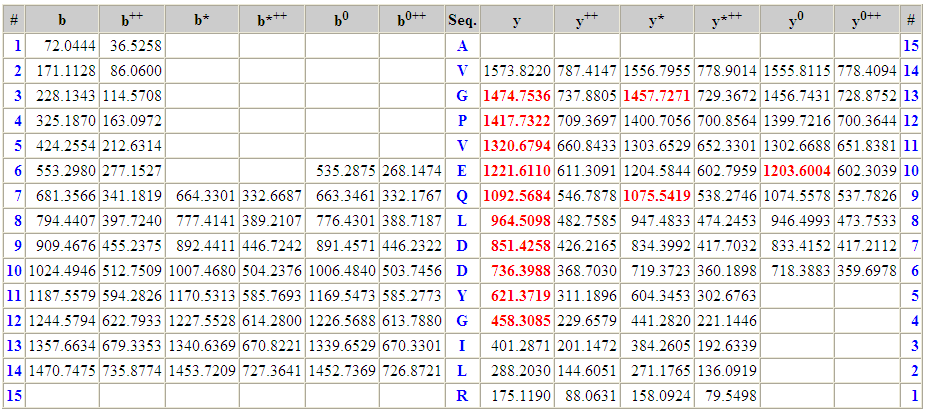


**(B)**

**
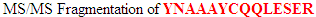
**

**
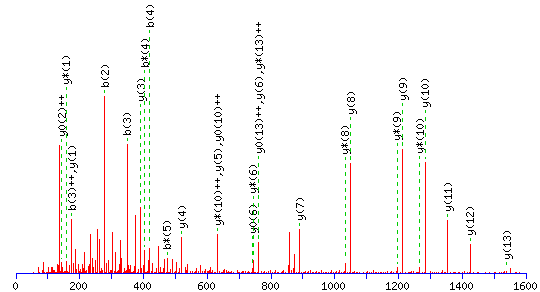
**

**
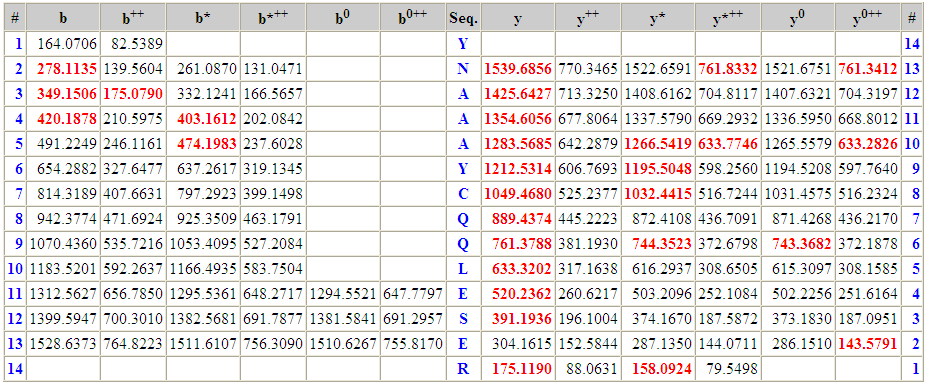
**

**
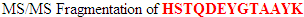
**

**
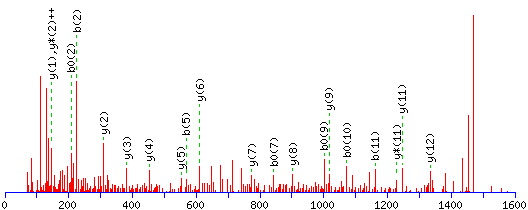

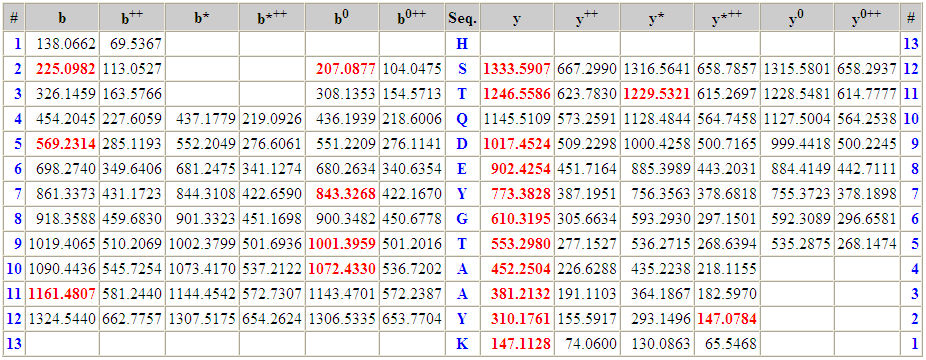
**


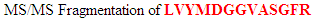


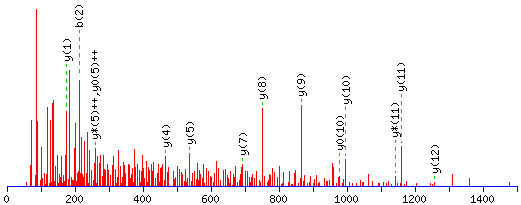


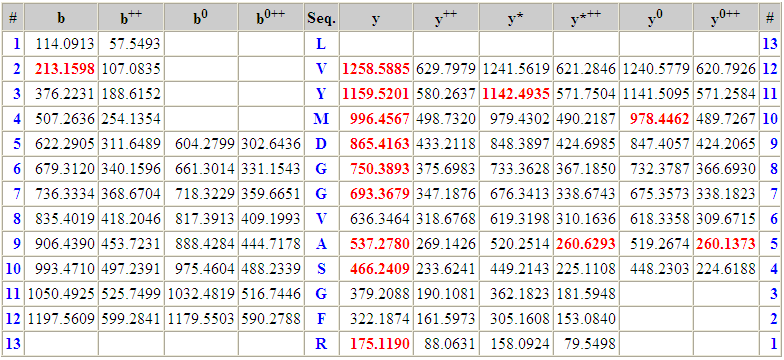


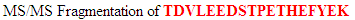


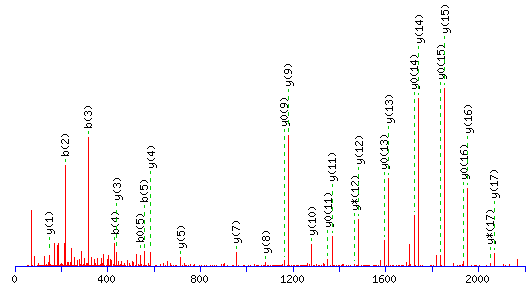

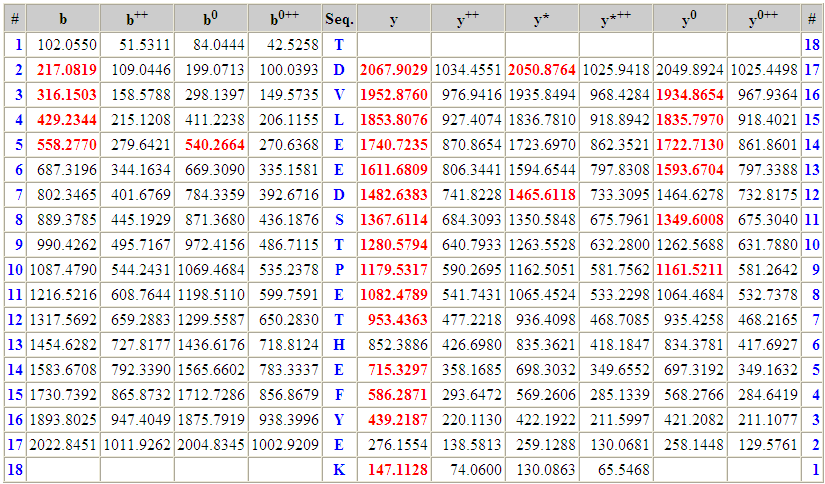


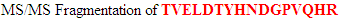

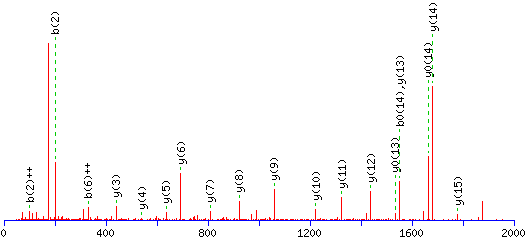

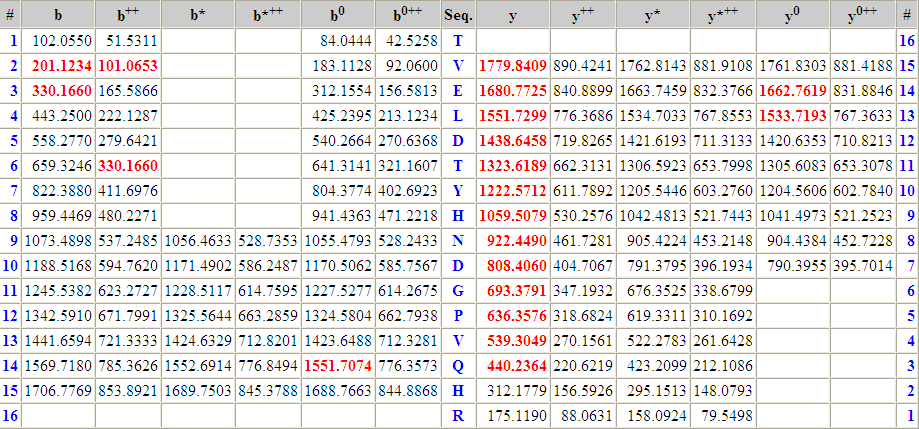


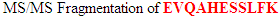

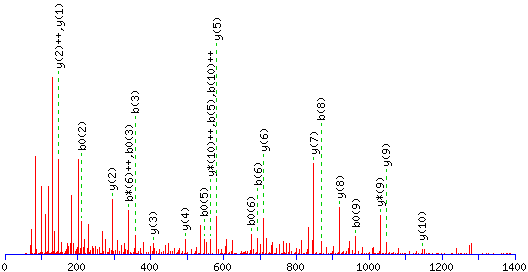

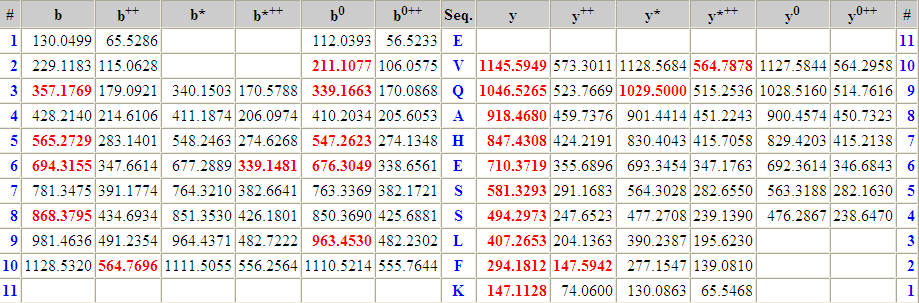


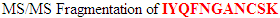

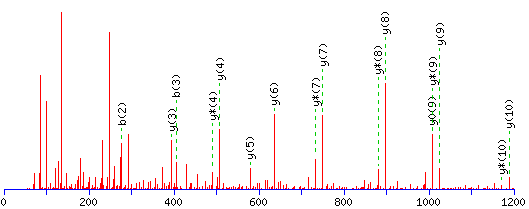

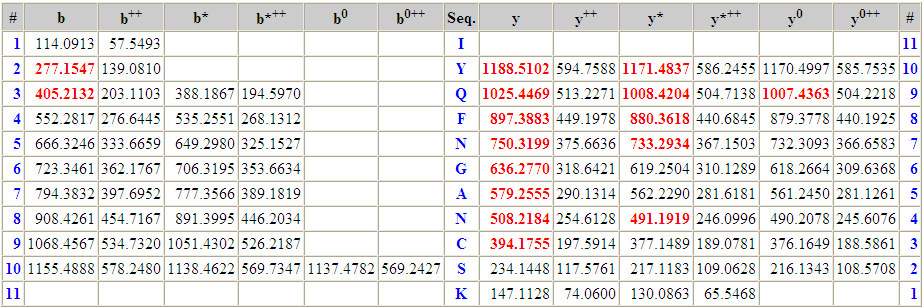


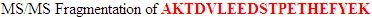

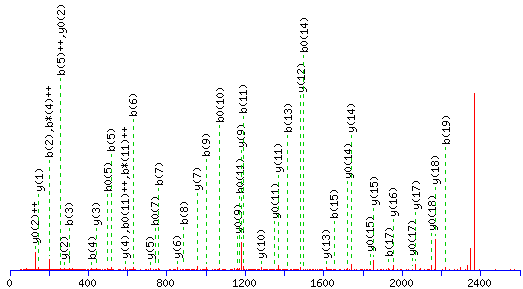

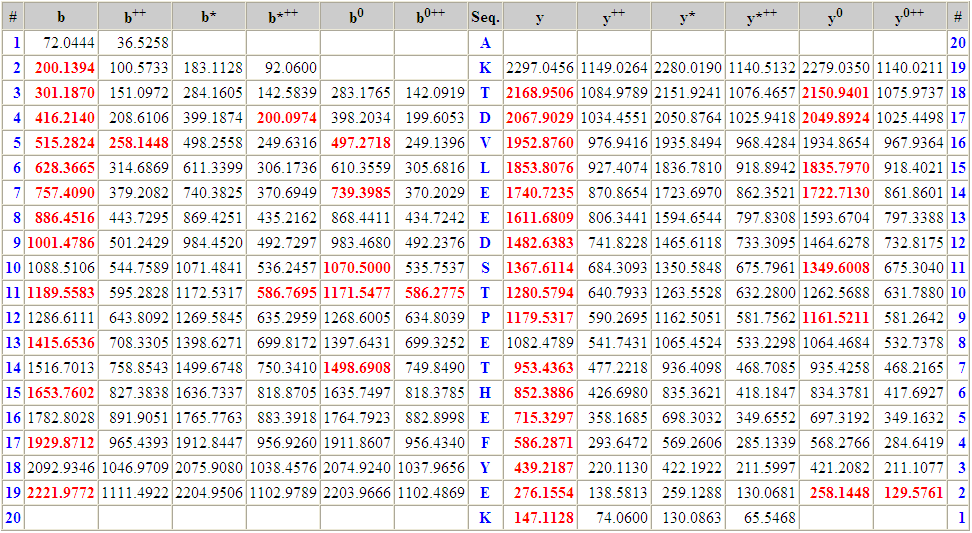


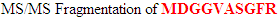

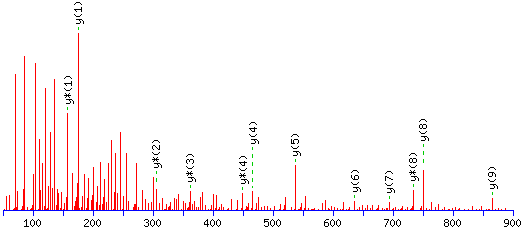


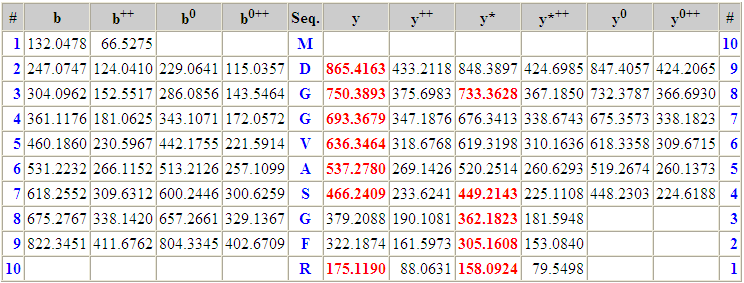

Supplement: Additional file 2 — Figure S1 - The MS/MS spectra of unique peptides that matched to (A) MPPbeta and (B) severin. [file 1477-5956-8-25-S2.doc]
